# Comprehensive analysis and validation of angiogenesis in vascular dementia from the perspective of diagnosis, prevention, and treatment

**DOI:** 10.3389/fgene.2025.1646991

**Published:** 2025-09-08

**Authors:** Lijun Xu, Yujiao Wang, Daojun Xie

**Affiliations:** ^1^ Hefei High-Tech Cardiovascular Hospital, Hefei, Anhui, China; ^2^ Anhui University of Chinese Medicine, Hefei, Anhui, China; ^3^ Encephalopathy Center, The First Affiliated Hospital of Anhui University of Chinese Medicine, Hefei, Anhui, China

**Keywords:** vascular dementia, angiogenesis, bioinformatics, snRNA-seq analysis, experimental verification

## Abstract

**Background:**

Angiogenesis is a critical pathological process in vascular dementia (VD), yet current therapeutic strategies targeting this mechanism remain limited. Identifying novel molecular pathways involved in angiogenesis holds significant promise for advancing both diagnostic and therapeutic approaches for VD.

**Methods:**

We first applied weighted gene coexpression network analysis (WGCNA) and differentially expressed gene (DEG) analysis, combined with phenotypic gene database mining, to identify angiogenesis-associated genes in VD. We then used the Least Absolute Shrinkage and Selection Operator (LASSO) regression to select key diagnostic genes. The diagnostic efficacy of these genes was evaluated using receiver operating characteristic (ROC) curve analysis, while their association with immune cell infiltration was assessed via xCell immunoinfiltration. Using single-nucleus RNA sequencing (snRNA-seq), we determined the cellular distribution of key genes and applied Gene Set Enrichment Analysis (GSEA) to analyze functional pathways in the differentially expressed cell clusters. Finally, we validated gene expression changes in the hippocampus of bilateral common carotid artery occlusion (BCCAO)-induced VD rats using quantitative polymerase chain reaction (qPCR) and Western blot (WB).

**Results:**

Ultimately, we screened five key genes, namely, *CCL2*, *VEGFA*, *SPP1*, *ANGPT2*, and *ANGPTL4*, which were all downregulated in the BCCAO model. The results of snRNA-seq showed that key genes were mainly clustered in microglia, endothelial cells, and astrocytes. Microglia, endothelial cells, and astrocytes play a key role in regulating angiogenesis.

**Conclusion:**

These five key genes might be used as angiogenesis diagnostic genes for VD and might be novel potential targets for diagnosis, treatment, and prevention.

## 1 Introduction

Vascular dementia (VD) ranks as the second most common form of dementia after Alzheimer’s disease (AD), arising from cerebrovascular pathologies ranging from microvascular dysfunction to major vessel occlusion that collectively disrupt neuronal homeostasis. Epidemiological data reveal an age-dependent progression, with prevalence rates reaching 1.1% in populations over 50 years and demonstrating a characteristic 5-year doubling pattern (Cao et al., 2020). In North America and Europe, VD accounts for 15%–20% of all dementia cases, and this figure may rise to 30% in Asia (Fu et al., 2023). Notably, the prevalence of VD is highest among dementia patients under 50 years of age (Hendriks et al., 2021). Risk factors for VD include poor diet, obesity, hypercholesterolemia, high blood pressure, and persistent heavy alcohol consumption. Furthermore, 79.4% of individuals diagnosed with VD had two or more comorbidities, which poses a significant burden on the elderly population (Morgan and Mc Auley, 2024). The complete pathogenesis of VD is complex and multifactorial, and remains to be fully elucidated. Despite this uncertainty, there exists a degree of mechanistic commonality among the various subtypes (Prajjwal et al., 2023). Vascular lesions are linked to several biological events that may lead to VD (Bir et al., 2021). Current therapeutic strategies, including cholinesterase inhibitors and cerebroactive agents, offer limited symptomatic relief without addressing disease progression. This therapeutic gap underscores the critical need for mechanism-based diagnostic biomarkers and targeted interventions that may enable early intervention and potentially modify disease course.

The hallmark pathological feature of VD is chronic cerebral hypoperfusion (CCH), which induces progressive neuronal ischemia and hypoxia, ultimately driving memory impairment and cognitive decline (Ghajar et al., 2006). Given this pathophysiology, timely restoration of cerebral blood flow represents a promising therapeutic strategy for VD. Angiogenesis, a tightly regulated physiological process, involves sprouting, proliferation, migration, and remodeling of endothelial cells from pre-existing vasculature to form new functional microvascular networks (Yang et al., 2013). In VD, cerebral hypoperfusion-induced ischemia disrupts neuronal homeostasis, making therapeutic angiogenesis a critical mechanism for restoring tissue perfusion, promoting neurogenesis, and facilitating neural repair (Xiao et al., 2019). Growing evidence suggests that enhancing hippocampal angiogenesis improves spatial learning and memory in preclinical VD models (Xiao et al., 2019; Han et al., 2022; Yang et al., 2017). Importantly, angiogenesis not only ameliorates ischemic damage but also provides structural and functional support for synaptic reorganization and neural circuit repair. Thus, modulating angiogenesis represents a key therapeutic avenue for improving functional recovery and long-term prognosis in VD. CCH represents an established pathological contributor to AD (Duncombe et al., 2017; De la Torre, 2021). Prior studies have characterized angiogenesis-related diagnostic signatures in AD, notably implicating *PLCB1* in regulating neovascularization through MAPK and Ca^2+^ signaling pathways (Wang et al., 2022; Jara-Medina et al., 2024). In contrast, the molecular mechanisms governing angiogenesis in vascular dementia (VD) remain poorly defined.

Early-stage VD is often clinically silent, underscoring the critical need for robust angiogenesis-related biomarkers to enable timely diagnosis and intervention. Integrative bioinformatics approaches provide a powerful platform for deciphering disease mechanisms at cellular and molecular levels, with particular utility in identifying transcriptomic signatures with diagnostic and therapeutic potential (Altmann, 2018). While angiogenesis dysfunction is increasingly recognized as a key pathological driver in VD, the precise molecular mediators and regulatory networks remain poorly characterized. To address this knowledge gap, we leveraged multi-omics bioinformatics combined with single-nucleus RNA sequencing (snRNA-seq) to systematically identify VD-specific angiogenic biomarkers and delineate their underlying molecular mechanisms. This comprehensive approach not only reveals novel therapeutic targets but also provides a mechanism-guided framework for developing precision interventions in VD management.

## 2 Materials and methods

**FIGURE 1:**
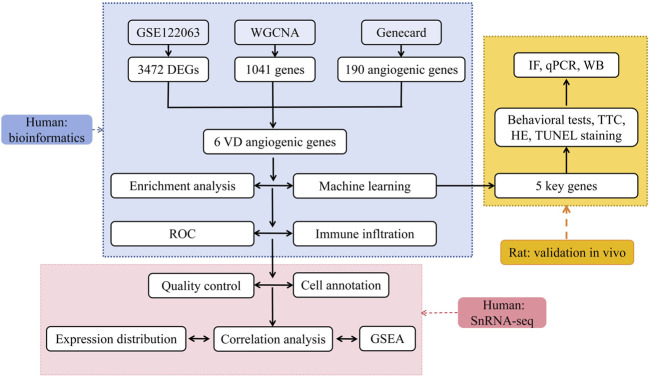
Flow chart of research design.

The detailed working flow chart is shown in Figure 1.

### 2.1 Reagents and drugs

For more information about the reagents used in this study, see (Supplementary Table S1).

### 2.2 Rat

Eight-week-old male Wistar rats (body weight: 200–220 g) were obtained from Hefei Qingyuan Biotechnology Co., Ltd (Hefei, China). The animals were maintained under standard laboratory conditions with a 12-h light/dark cycle and *ad libitum* access to food and water. All experimental procedures were performed in strict accordance with the National Institutes of Health Guide for the Care and Use of Laboratory Animals and were approved by the Institutional Animal Care and Use Committee of the First Affiliated Hospital of Anhui University of Chinese Medicine (Approval No.: AZYFY-2024-1002).

### 2.3 Source of data

Public datasets were identified through structured GEO queries using angiogenesis- and VD-specific keywords (Supplementary Table S3A). Final dataset selection prioritized sample size, platform uniformity, and clinical annotation completeness.

RNA-seq transcriptome data were retrieved from the Gene Expression Omnibus (GEO) database (accession: GSE122063), comprising 44 healthy controls (HC) and 36 VD samples (McKay et al., 2019). The cohort demographics showed comparable age distributions between groups (HC: 78.82 ± X years; VD: 80.89 ± X years; mean difference: −2.07 ± 2.09 years, p = 0.26 by t-test; χ^2^ p > 0.99), with detailed statistics presented in Supplementary Figure S2 and Supplementary Table S3B.

For single-nucleus resolution analysis, we acquired the GSE213897 dataset from GEO, containing 13 samples with annotated microglia and endothelial cell populations (Mitroi et al., 2022). The cohort demographics showed comparable age distributions between groups (HC: 82.60 ± X years; VD: 80.50 ± X years; mean difference: −2.10 ± 3.23 years, p = 0.5290 by t-test). Sex distribution data were presented in Supplementary Table S3C. Additionally, all VD samples in this cohort underwent comprehensive neuropathological examination to exclude AD pathology (including Aβ deposition) and other dementia-related co-pathologies (Mitroi et al., 2022). This dataset yielded 31,240 nuclei from HC and 51,404 from VD subjects after quality control.

Additionally, angiogenesis-related genes were curated from the GeneCards database (https://www.genecards.org) using a stringent relevance score threshold (>5), identifying 190 candidate genes for subsequent analysis.

### 2.4 Identification of VD angiogenesis phenotypic genes

We performed weighted gene co-expression network analysis (WGCNA) using R software (v4.3.3) to identify disease-associated gene modules. The analysis included (Cao et al., 2020): determination of the optimal soft threshold power (Fu et al., 2023), construction of a scale-free co-expression network, and (Hendriks et al., 2021) evaluation of module-trait relationships. Gene modules showing significant correlations with VD (p < 0.05) were selected for downstream analysis.

The R package “limma” (Ritchie et al., 2015) was employed to analyze the differential expression of the GSE122063 dataset. A threshold of P.adj <0.05 was established to define the differentially expressed genes (DEGs) associated with VD. For the visual analysis of DEGs, the software packages “ggplot2” and “pheatmap” were utilized to create volcano plots. Furthermore, the combination of VD module genes, DEGs, and angiogenesis gene sets was analyzed using the “VennDiagram” package to identify angiogenesis-related phenotypic genes.

### 2.5 Functional enrichment analysis and key genes identification

We performed functional enrichment analysis of VD angiogenesis-related phenotypic genes using Gene Ontology (GO) and Kyoto Encyclopedia of Genes and Genomes (KEGG) pathways. To identify diagnostic biomarkers, we applied machine learning approaches for preliminary gene screening. Using the LASSO (Least Absolute Shrinkage and Selection Operator) logistic regression algorithm implemented in the R package “glmnet,” we selected key genes by applying an L1-penalty (lambda) that shrinks less important variable coefficients to zero while retaining diagnostically relevant features (Xu et al., 2023). This approach enabled us to construct an optimal classification model while effectively reducing feature dimensionality. Furthermore, we evaluated the diagnostic value of key genes using the “pROC” package in R, plotted receiver operating characteristic (ROC) curves, and performed Wilcoxon rank sum tests to verify key gene expression levels.

### 2.6 Immune infiltration analysis

To further validate the results of our bioinformatics analysis and to explore the relationship between VD and immunity, we employed the “xCell” package for immunoinfiltration analysis. Utilizing the method of single-sample gene set enrichment analysis (ssGSEA), this package enables the examination of correlations between VD and various immune cell types based on the gene expression data of 64 immune and stromal cells. Additionally, we investigated the correlation between key genes associated with VD angiogenesis and the levels of immune infiltration, with * indicating statistically significant differences (P < 0.05).

### 2.7 snRNA-seq analysis

To understand the characteristics of VD snRNA-seq, we analyzed dataset GSE213897 following the standard “Seurat” process (Butler et al., 2018). We eliminated cells expressing fewer than 300 or more than 7000 genes, as well as those with mitochondrial content exceeding 10% and erythrocyte content above 3%. The samples were combined and de-batched using the R package “harmony” (Korsunsky et al., 2019). The “FindVariableFeatures” function was employed to identify the first 3000 highly variable expressed genes. Dimensionality reduction, clustering, and visualization of clusters were performed using the RunUMAP function in Seurat (Becht et al., 2018). To identify marker genes in each cell cluster, we utilized the “FindAllMarkers” function along with the “wilcox” test, setting logfc.threshold = 0.25 and P.val.adj <0.05 as screening criteria. Subsequently, the identified marker genes were input into the CellMarker 2.0 database (http://117.50.127.228/CellMarker/CellMarker_annotation.jsp) for cell type annotations (Hu et al., 2023). The characteristic marker genes provided by the website were then used for verification. The “scCustomize” package, in conjunction with the “ggplot2” package, was employed to display the expression of key genes in each cell cluster. For cell clusters exhibiting high expression of key genes, we utilized the FindMarkers function, setting logfc. Threshold = 0.5 and P.adj <0.05 as thresholds to calculate DEGs in the HC and VD groups of each cell cluster, followed by GSEA enrichment analysis to uncover the common biological functions of each cell cluster.

### 2.8 VD model construction

#### 2.8.1 Grouping and model

Thirty Wistar rats were randomly assigned to sham-operated (Sham) or VD model groups (n = 15/group). After 12-h fasting (water *ad libitum*), the animals were anesthetized by intraperitoneal injection of 2.5% tribromoethanol (6 mL/kg, Tigergene, Lot No.: 2412). The VD model was established by permanent bilateral common carotid artery ligation: following neck midline incision and blunt dissection, both arteries were sequentially ligated using surgical thread. Successful model induction was confirmed by ipsilateral ptosis and palpebral fissure reduction post-anesthesia. Brain tissues were collected for analysis after euthanasia, with carcass disposal following institutional guidelines.

#### 2.8.2 Behavioral tests

The spatial learning and memory abilities of rats were evaluated using the Morris water maze test at 4 weeks post-surgery. Six rats per group were randomly selected for testing in a circular pool divided into four quadrants, with a hidden platform (9 cm diameter, 2 cm below the water surface) placed in quadrant I. The 6-day protocol included 5 days of acquisition trials (4 trials/day) followed by a probe test on day 6. All experiments were conducted in a controlled environment with minimal disturbances. (n = 6/group).

### 2.9 Tissue collection and 2, 3, 5-triphenyltetrazolium chloride (TTC) staining

After behavioral testing, the rats were deeply anesthetized by intraperitoneal injection of 2.5% tribromoethanol (6 mL/kg). We performed cardiac perfusion by inserting a needle into the left ventricle while creating an outflow incision in the right atrium. We perfused ice-cold 0.9% saline (∼200 mL/rat) until achieving complete organ blanching. The rats were humanely euthanized via deep anesthesia followed by transcardial perfusion fixation, which ensures rapid and painless death while preserving tissues for further analysis. This method complies with standard ethical guidelines for laboratory animal euthanasia, as it first induces unconsciousness (via anesthesia) before performing perfusion, preventing any potential distress. We then rapidly extracted the brains, carefully dissected the hippocampi, flash-froze them in liquid nitrogen, and stored the samples at −80 °C for subsequent analysis.

For infarct quantification, coronal sections (2 mm thick) were incubated in 2% TTC (37 °C, 30 min, dark), then PBS-washed (3×). Viable tissue stained red while infarcts remained pale. Three randomly selected brains per group were analyzed.

### 2.10 Histopathological assessment by hematoxylin-eosin (HE) staining

According to the anesthesia protocol described in Section 2.9, the rats were deeply anesthetized and then subjected to transcardial perfusion with 4% paraformaldehyde (PFA) until the visceral tissues turned pale, indicating successful perfusion. We randomly selected brain tissue samples from three rats per group for histological analysis. Subsequently, tissue sections with a thickness of 3–5 μm were prepared and subjected to HE staining. After mounting, pathological observations of the hippocampal region and cerebral cortex were conducted under an optical microscope, and photomicrographs were captured for documentation. (n = 3 rats/group).

### 2.11 TUNEL staining for detection of neuronal apoptosis

Brain tissue sections were prepared as described in Section 2. 10, with three randomly selected rats used as experimental samples. The TUNEL assay was performed strictly following the manufacturer’s protocol. After rinsing, dehydration, and mounting, we observed the samples under a fluorescence microscope. To ensure data representativeness, at least three randomly selected fields of view (FOVs) per section were captured for analysis. (n = 3 rats/group).

### 2.12 Immunofluorescence staining for CD31 protein expression

Following the manufacturer’s instructions, brain sections were dewaxed and subjected to antigen retrieval. The sections were then blocked with PBS containing 3% donkey serum and 0.3% Triton X-100 for 1 h at room temperature, followed by incubation with the primary antibody against CD31/PECAM1 (1:200 dilution, BOSTER) overnight at 4 °C. After washing, the CD31/PECAM1-labeled sections were incubated with the secondary antibody for 1 h at room temperature. Finally, the sections were rinsed with PBS and counterstained with 4′,6-diamidino-2-phenylindole (DAPI). Semi-quantitative analysis of fluorescence intensity was performed using ImageJ software. (n = 3 rats/group).

### 2.13 qPCR detection

We carefully weighed approximately 20 mg of hippocampal tissue and transferred it to a pre-cooled 1.5 mL EP tube. We homogenized the tissue in lysis buffer at −20 °C using a mechanical homogenizer. Following the manufacturer’s protocol, we extracted total RNA through column purification with *GAPDH* serving as our internal reference control. We then performed reverse transcription to generate cDNA templates. Using quantitative RT-PCR, we measured and analyzed the expression levels of angiogenesis-related genes, including *CCL2*, *VEGFA*, *SPP1*, *ANGPT2*, *ANGPTL4*, *CD31*, and *HIF-1α*. The sequences of the primers are detailed in Supplementary Table S4.

### 2.14 Western blot (WB) analysis

We extracted proteins from frozen hippocampal tissues using RIPA buffer with protease/phosphatase inhibitors. After homogenization and centrifugation (12,000 × g, 5 min, 4 °C), we quantified protein concentrations by BCA assay. For each sample, 20 μg of total protein was loaded and separated by SDS-PAGE, followed by electrophoretic transfer onto 0.45 μm PVDF membranes. The membranes were then blocked with rapid blocking buffer for 20 min and subsequently incubated overnight at 4 °C with the following primary antibodies: CD31/PECAM1 (BOSTER, A01513-3, 1:2000), HIF-1α (BOSTER, PB9253, 1:2000), MCP-1/CCL2 (BOSTER, BA1843-2, 1:2000), VEGFA (BOSTER, BA0407, 1:2000), SPP1 (BOSTER, PB0589, 1:2000), ANGPT2 (BOSTER, A00370-2, 1:2000), ANGPTL4 (Proteintech, 18374-1-AP, 1:1000), and Tubulin (BOSTER, BM4273, 1:100000). After secondary antibody incubation (HRP-conjugated, 2 h, RT), we detected bands by ECL and quantified using ImageJ, normalizing to Tubulin.

### 2.15 Statistical analysis

Statistical analyses were performed using GraphPad Prism software (version 8. 0). Continuous variables are presented as mean ± standard deviation (mean ± SD). For multi-group comparisons, we applied either one-way ANOVA (for normally distributed data) or the Kruskal–Wallis test (for non-parametric data), followed by appropriate *post hoc* tests when significant differences were detected. Two-group comparisons were analyzed using independent samples t-tests for parametric data or Mann-Whitney U tests for non-parametric data. A p-value <0.05 was considered statistically significant.

## 3 Results

### 3.1 Acquisition of VD angiogenesis genes

**FIGURE 2:**
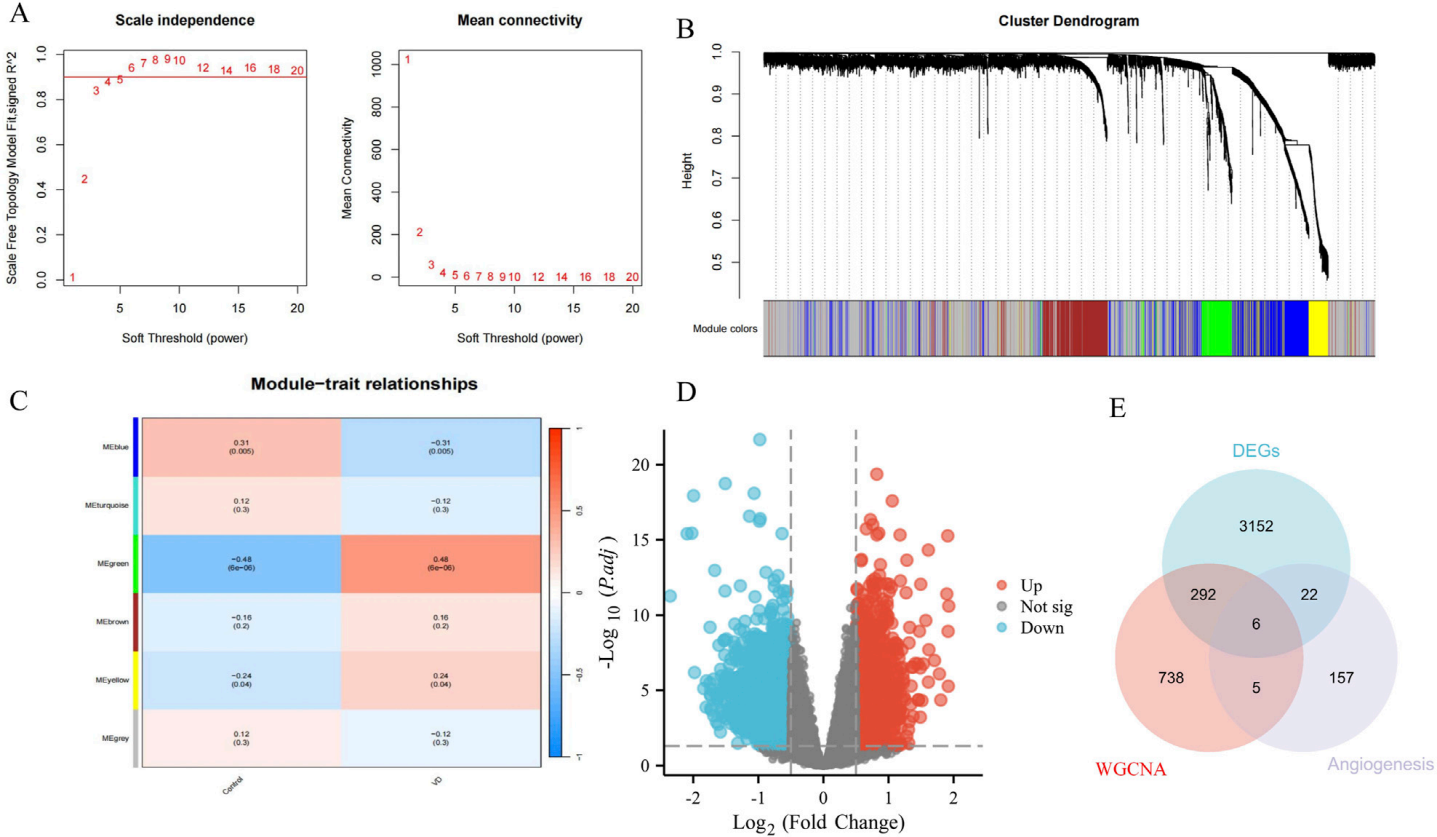
Identification of VD angiogenesis phenotypic genes. **(A)** Soft threshold. **(B)** Cluster dendrogram. **(C)** Module-trait relationships. **(D)** Volcano plots of DEGs from GSE122063. **(E)** Venn diagram of DEGs, WGCNA, and angiogenesis genes.

A total of 1041 VD signature genes from green/blue modules were identified by WGCNA (soft threshold power = 5) to define the module genes with p < 0.05 that were statistically significant and associated with VD pathogenesis (Figures 2A–C). Based on data set GSE122063, with |logFC| > 0.5 and P.adj <0.05 as DEGs, 3472 DEGs between HC and VD were screened, including 1541 upregulated genes and 1931 downregulated genes (Figure 2D). We intersected the genes of WGCNA and DEGs with the angiogenesis phenotype genes obtained from the GeneCards database, and obtained 6 VD angiogenesis phenotypic genes (Figure 2E).

### 3.2 Enrichment analysis, machine learning, and ROC

**FIGURE 3:**
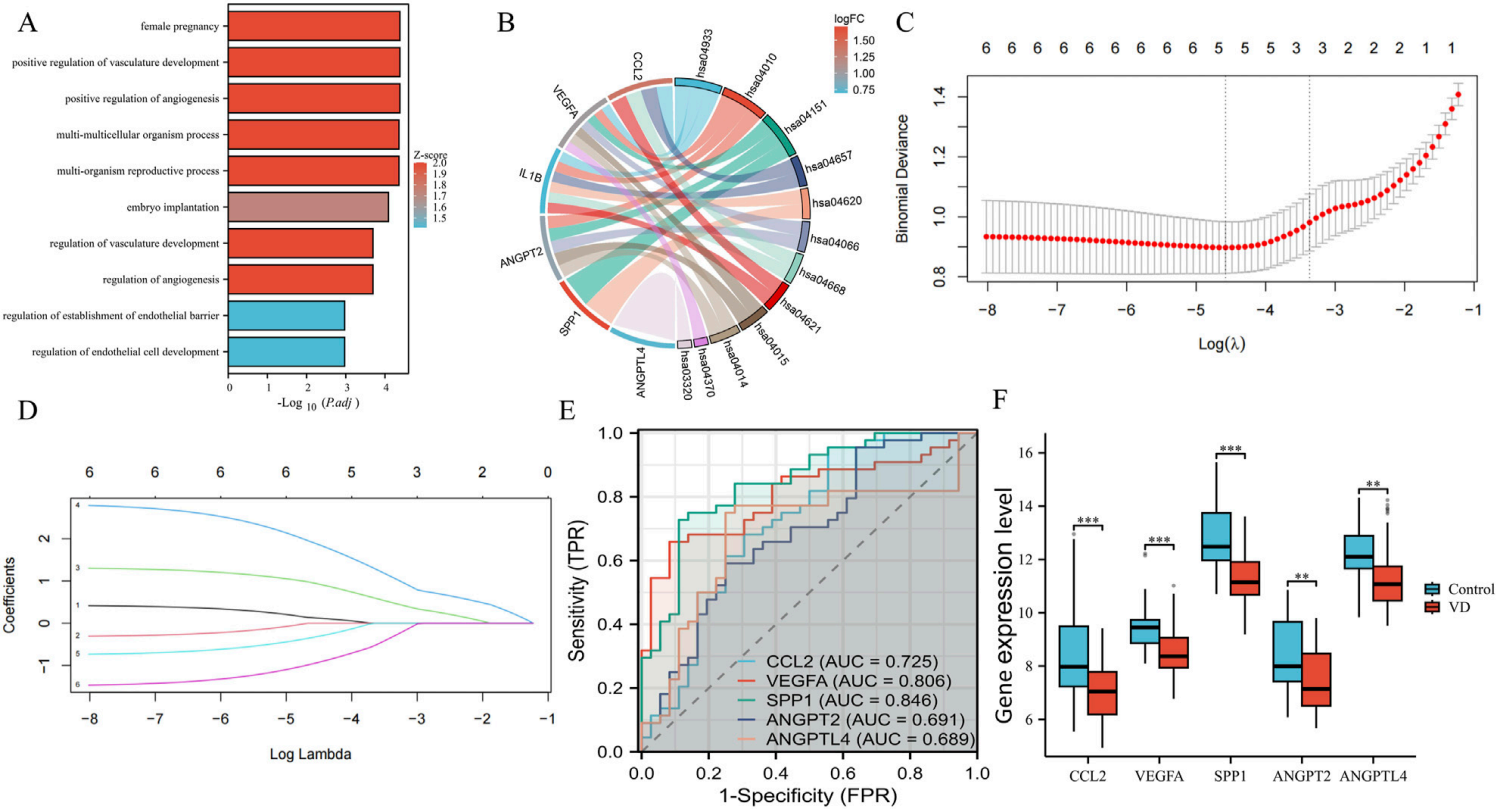
Enrichment analysis, key genes acquisition, and verification. **(A)** The top 10 columns in the enrichment analysis in GO: BP. **(B)** The main signaling pathways of KEGG enrichment. **(C)** Cross-verification curve. **(D)** LASSO coefficient path map of 6 phenotypic genes. **(E)** ROC curves of phenotypic key genes in GSE122063. **(F)** Validation of expression levels in GSE122063.

The obtained 6 VD angiogenesis phenotypic genes were enriched. Using P. value <0.05 as a threshold, we identified 678 GO-BP terms and 36 KEGG pathways. The top 10 results are shown in Figure 3A. The top 10 results of GO: BP enrichment analysis are positive regulation of angiogenesis, positive regulation of vasculature development, female pregnancy, multi-organism reproductive process, multi-multicellular organism process, embryo implantation, regulation of angiogenesis, regulation of vasculature development, regulation of endothelial cell development, and regulation of establishment of endothelial barrier. It can be seen that the main enrichment results are concentrated in the regulation of angiogenesis, endothelial cell (EC) development, and vasculature development. The main signaling pathways of KEGG enrichment are the AGE-RAGE signaling pathway in diabetic complications, the MAPK signaling pathway, PI3K-Akt signaling pathway, IL-17 signaling pathway, Toll-like receptor signaling pathway, HIF-1 signaling pathway, TNF signaling pathway, NOD-like receptor signaling pathway, Rap1 signaling pathway, and Ras signaling pathway (Figure 3B), which are critical for the regulation of angiogenesis. In order to further test the diagnostic value of these 6 phenotypic genes, we used the LASSO regression algorithm. The optimal lambda (λ = 5) was selected via 10-fold cross-validation, finally obtaining 5 genes as phenotypic key genes (Figures 3C,D). To verify the diagnostic value of these 5 highly expressed genes, we verified the diagnostic value of the original dataset through ROC, and found that *CCL2*, *VEGFA* and *SPP1* had high diagnostic value, while *ANGPT2* and *ANGPTL4* had moderate diagnostic value (Figure 3E). They were all downregulated in VD transcriptome data sets, and the difference was statistically significant (Figure 3F).

### 3.3 Immune infiltration analysis

**FIGURE 4:**
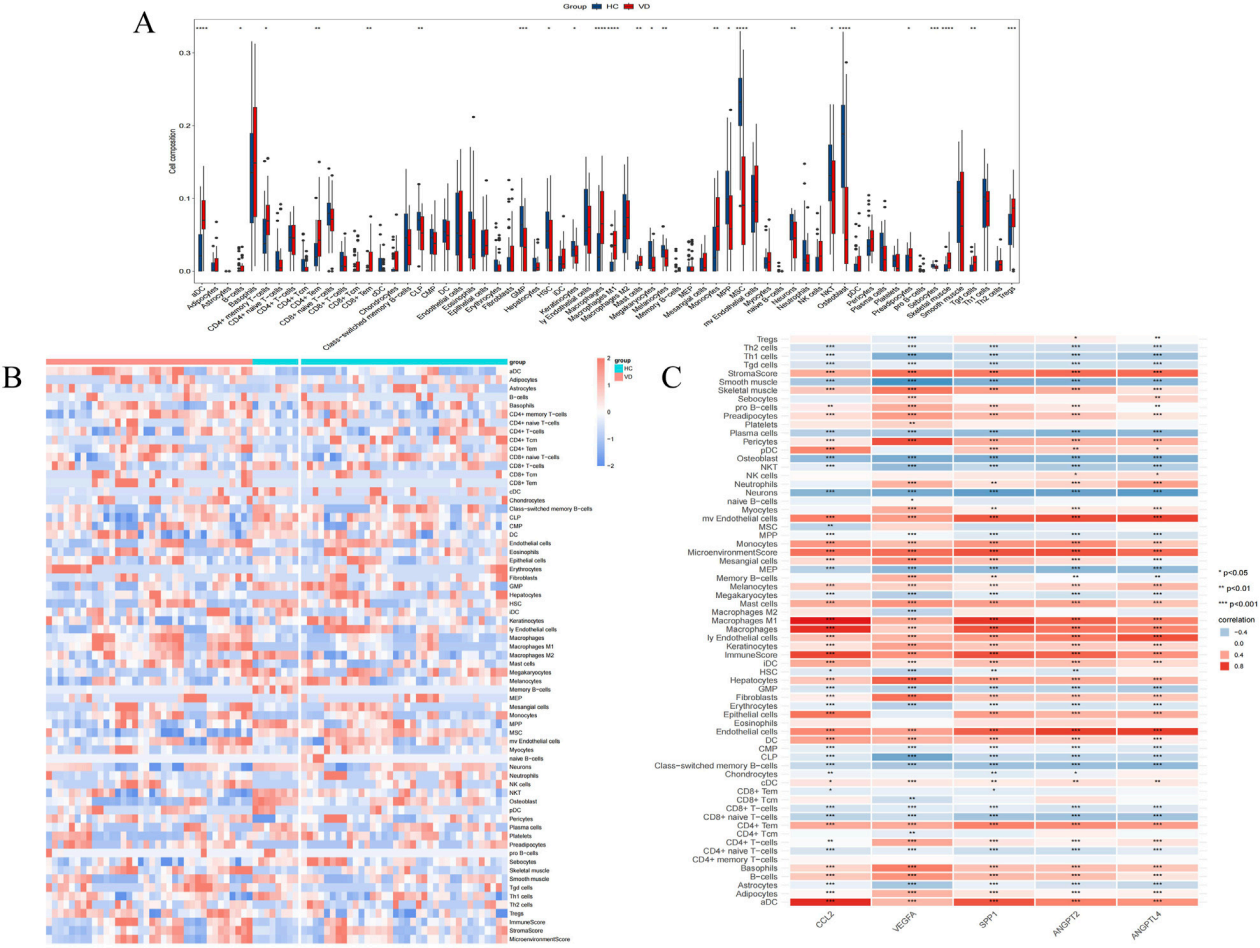
Immune cells infiltration analysis. **(A)** Comparison of the scores for immune cells estimated by the xCell algorithm between the VD and HC groups in GSE122063. **(B)** Heatmap of immunoinfiltration in GSE122063 in the VD and HC groups. **(C)** Infiltration correlation between key genes and 64 kinds of immune cells in GSE122063.

Considering the correlation between VD and immune cells, we performed immune infiltration analysis. In 64 kinds of immune cells, there are 26 kinds of cell types in the significant differences between VD group and control group (P < 0.001 ∼ “* * *,” P < 0.01 ∼ “* *,” P < 0.05 ∼ “*”) (Figure 4A), and in VD group, aDC, Adipocytes, B cells, CD4^+^ memory T-cells, CD4^+^ Tem, CD8^+^ Tem, Macrophages, Macrophages M1, Mast cells, Monocytes, MPP, Preadipocytes, Skeletal muscle, Tgd cells, Tregs increased expression, immune cell expression of CLP, GMP, HSC, Keratinocytes, Megakaryocytes, Melanocytes, MSC, Neurons, NKT, Osteoblast, Sebocytes were reduced (Figure 4B). It can be seen that VD group and HC group have certain infiltration with different immune cells. In addition, we also evaluated the immune infiltration relationship between VD angiogenesis key genes and 64 types of immune cells, indicating that each phenotype gene has a very strong infiltration relationship with different immune cells (Figure 4C).

### 3.4 snRNA-seq analysis

**FIGURE 5:**
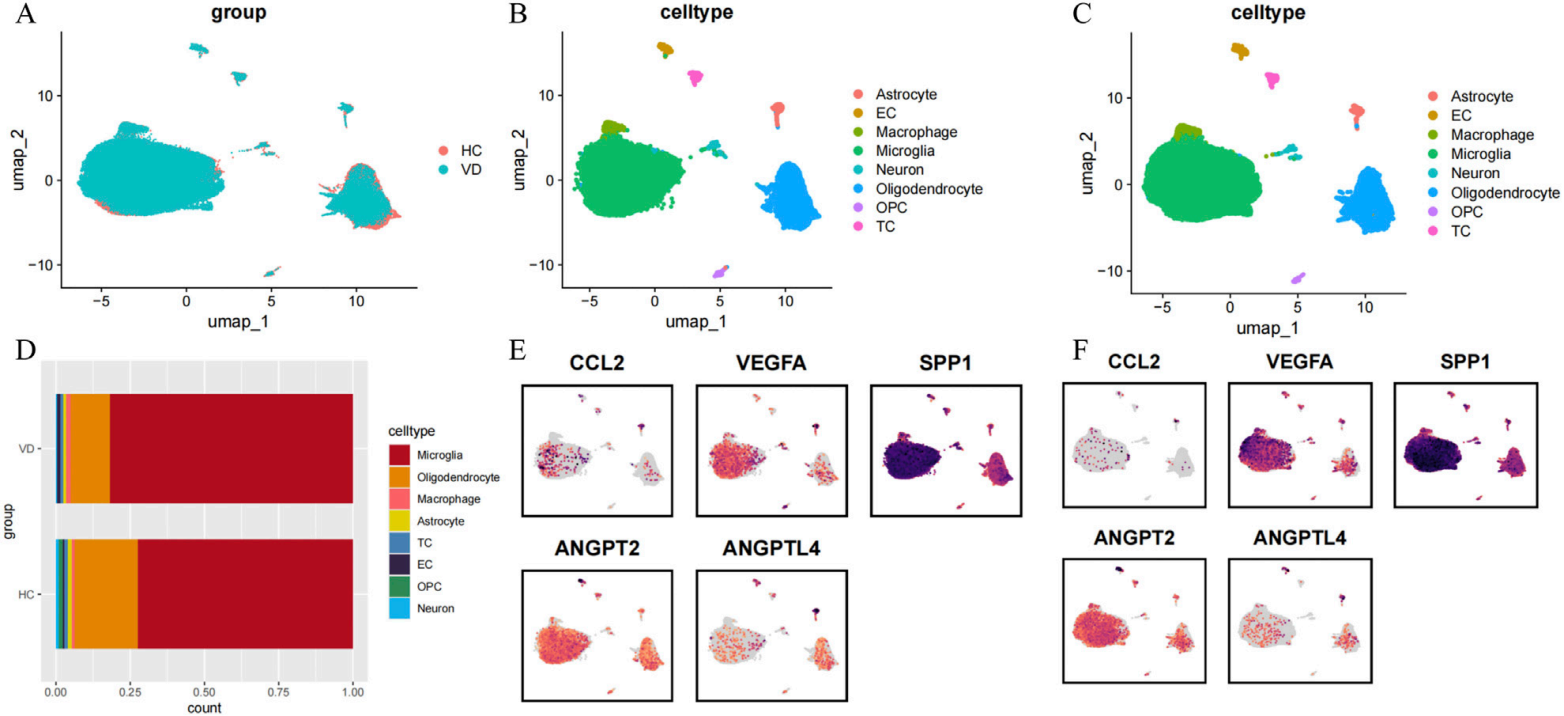
SnRNA-seq analysis. **(A)** Cluster distribution of VD and HC groups after data quality control. **(B)** The cellular annotation data for the HC group. **(C)** The cellular annotation data for the VD group. **(D)** The proportion of different cell types in the VD and HC groups. **(E)** The expression distribution of 5 key genes in the HC group. **(F)** The expression distribution of 5 key genes in the VD group.

To elucidate the transcriptomic landscape of key angiogenesis-related genes across various brain cell types, we conducted snRNA-seq analysis. The figures of snRNA-seq data after quality control are shown in the Supplementary Figure S5. Following Harmony batch effect correction, a significant reduction in batch effects was observed across all samples (Supplementary Figure S6). Figure 5A illustrates the distribution of cells following quality control, revealing that the two sample groups are well integrated within distinct cell clusters. Cluster analysis categorized the cells into nine clusters. CellMarker 2.0 was employed for cell annotation, identifying eight distinct cell types: Microglia, Oligodendrocyte, Macrophage, Astrocyte, T Cell (TC), Endothelial Cell (EC), Oligodendrocyte Precursor Cell (OPC), and Neuron. The cellular annotation data for the HC and VD groups are presented in Figures 5B,C, respectively. The mark gene validation plots for each cell cluster are shown in Supplementary Figure S7. Subsequently, we analyzed the cellular proportions within the samples (Figure 5D). Compared to the HC group, the proportion of oligodendrocyte, astrocyte, T cell, OPC, and neuron was found to decrease in VD tissues, while the proportions of microglia, macrophage, and endothelial cell increased. This suggests an imbalance among various brain cell types in the VD brain tissues. To further investigate the expression distribution of key genes, we calculated the average expression levels of these genes within the snRNA-seq dataset, revealing that key genes were broadly expressed in microglia, EC, and astrocytes, with notable differential expression (Figures 5E,F depict the expression distribution for the HC and VD groups, respectively). These 3 cell types are crucial for the regulation of angiogenesis.

### 3.5 GSEA enrichment analysis and intercellular correlation analysis

**FIGURE 6:**
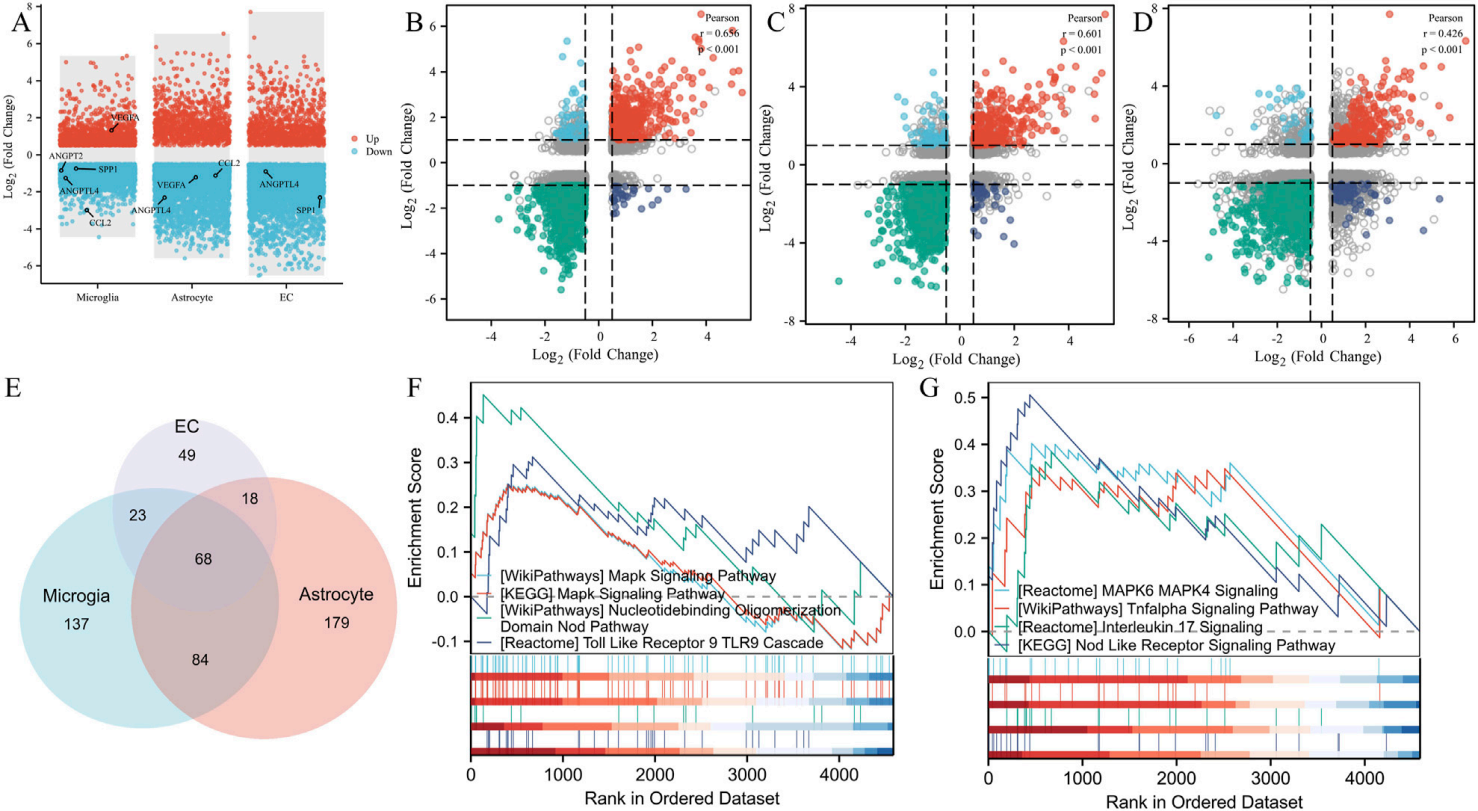
GSEA enrichment analysis and intercellular correlation analysis. **(A)** DEGs, volcanomap, and key genes distribution in microglia, astrocytes, and endothelial cells. **(B)** The correlation coefficient of DEGs between microglia and astrocytes. **(C)** The correlation coefficient of DEGs between microglia and EC. **(D)** The correlation coefficient of DEGs between astrocyte and EC. **(E)** Venn diagram of the GSEA pathway intersection of microglia, astrocyte, and EC. **(F)** Angiogenesis pathway demonstration of angiogenesis phenotypic genes. **(G)** Angiogenesis pathway demonstration in GSEA enrichment analysis.

To demonstrate the expression changes of these five key genes in different cell clusters, we performed DEGs visualization (Figure 6A). To clarify the functions of these 3 cells, we analyzed the correlation of DEGs between VD in microglia, endothelial cell and astrocyte, found that *CCL2*, *SPP1*, *ANGPT2* and *ANGPTL4* were downregulated in microglia, while *VEGFA* was upregulated in microglia. The expression of *CCL2*, *VEGFA,* and *ANGPTL4* was downregulated in astrocytes. The expression of *SPP1* and *ANGPTL4* in the endothelial cell was downregulated. These different expression changes of the five key genes in different cell clusters suggested that the 3 cell clusters might have synergistic effects. To analyze the correlation among the 3 cell types, we performed a correlation analysis and found that the correlation coefficient of DEGs between microglia and astrocyte was 0.656, which was statistically significant (Figure 6B). The correlation coefficient between microglia and EC was 0.601, also statistically significant (Figure 6C). Additionally, the correlation coefficient between astrocyte and EC was 0.426, with a statistically significant difference (Figure 6D). These results indicate a clear correlation in gene co-expression and function among the 3 cell types. GSEA enrichment analysis showed that 312 pathways were enriched in microglia, 349 in astrocyte, and 158 in EC (Figure 6E). The functional pathways of these three types of cells had a total of 68 intersections, and these intersections showed the same pathways as the angiogenesis phenotype genes (Figures 6F,G), which were mainly MAPK signaling pathway, NOD signaling pathway, TOLL Like signaling pathway, TL-17 signaling pathway, and HIF-1 signaling pathway.

### 3.6 Construction of animal models and analysis of pathological damage

**FIGURE 7:**
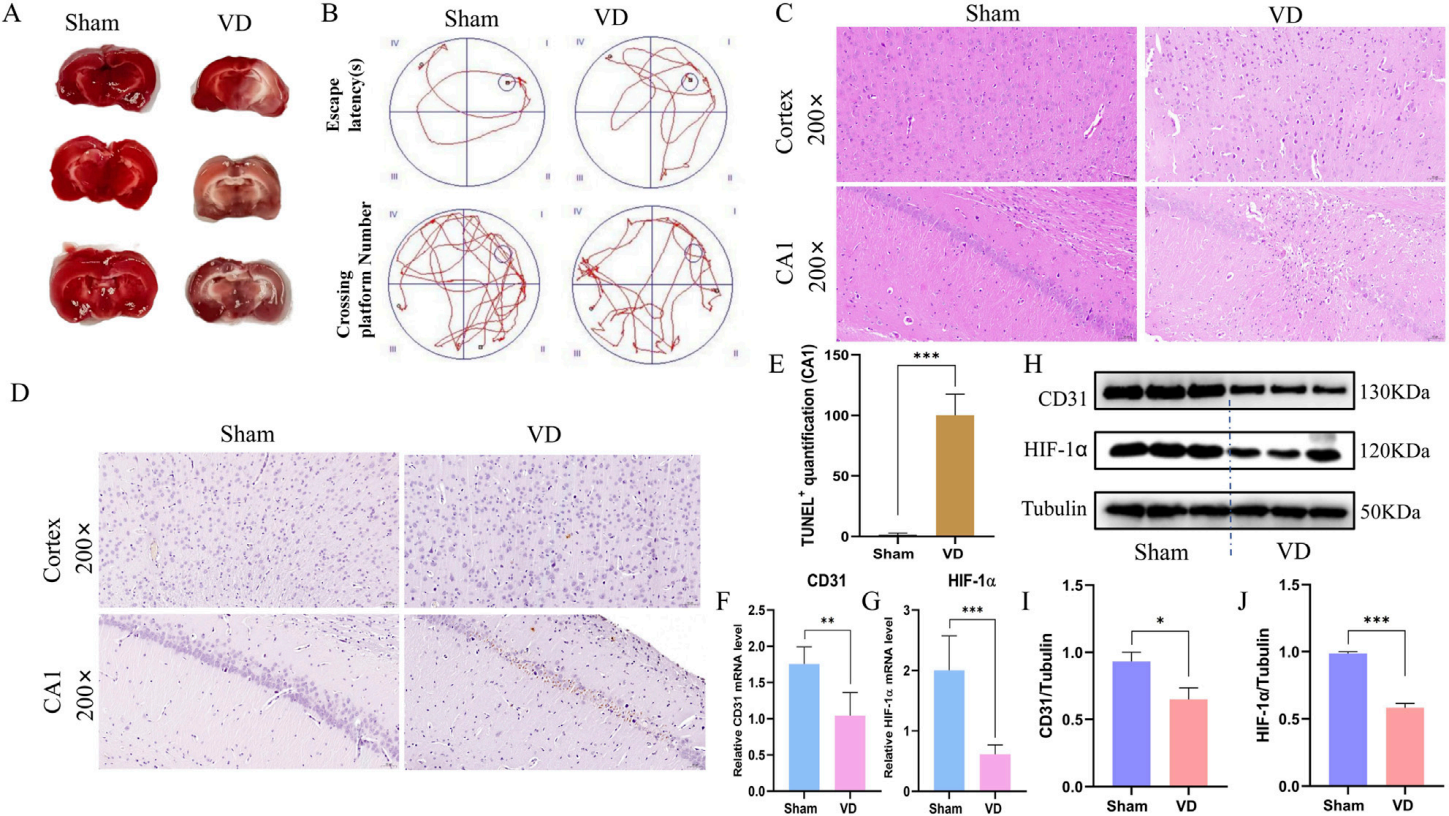
Construction of animal models and analysis of pathological damage. **(A)** TTC staining plots of the Sham and VD group. **(B)** Motion trajectory diagram. **(C)** Representative images stained with HE (scale bars = 50 μm). **(D)** Representative histopathological images obtained from TUNEL staining (scale bars = 50 μm), showing changes in the cortex hippocampus CA1. **(E)** Quantification of TUNEL+ cells in the hippocampal CA1 (n = 3). **(F)** Expression profile of CD31 between Sham and VD groups (qPCR). **P < 0.01. **(G)** Expression profile of HIF-1α between Sham and VD groups (qPCR; n = 6). ***P < 0.001. **(H)** WB strips. **(I)** Expression profile of CD31 between Sham and VD groups (WB). *P < 0.05. **(J)** Expression profile of HIF-1α between Sham and VD groups (WB; n = 3). ***P < 0.001.

The bilateral common carotid artery permanent occlusion (BCCAO) model can well simulate the environment in which VD occurs, so we subsequently used this model to verify the angiogenesis key genes of VD. After 4 weeks of modeling, TTC staining showed multiple cerebral cortex and subcortical infarcts in the VD group compared to the Sham group (Figure 7A). The Morris water maze test indicated that the escape latency of rats in the VD group was significantly prolonged (Supplementary Figure S8A). The frequency of crossing the platform was significantly reduced (Supplementary Figure S8B). The two groups of representative trajectory diagrams are shown in Figure 7B. To better characterize neuronal pathological changes in BCCAO rats, we conducted HE staining analysis. Histological examination revealed striking differences between the experimental groups. In sham controls, neurons exhibited normal morphology with tightly packed cell bodies, well-preserved nuclear structure, and absence of cytoplasmic vacuolization. In contrast, the BCCAO model group displayed marked neurodegenerative features, including pyknotic nuclei with nucleolar dissolution, disorganized cellular architecture, shrunken and deformed somata, along with abundant neuronal necrotic debris (Figure 7C). To further assess neuronal apoptosis, we performed TUNEL staining. Compared with the sham group, the BCCAO group exhibited significantly increased apoptotic activity in hippocampal neurons (Figures 7D,E). To further characterize angiogenesis-related phenotypic changes in the model group, we analyzed the expression of CD31 and HIF-1α at both transcriptional and protein levels using qPCR and Western blot (WB). The results demonstrated significant downregulated of CD31 and HIF-1α mRNA (Figures 7F,G) and corresponding protein levels (Figures 7H–J) in BCCAO model rats compared to sham controls.

### 3.7 Experimental validation of the key genes *in vivo*

**FIGURE 8:**
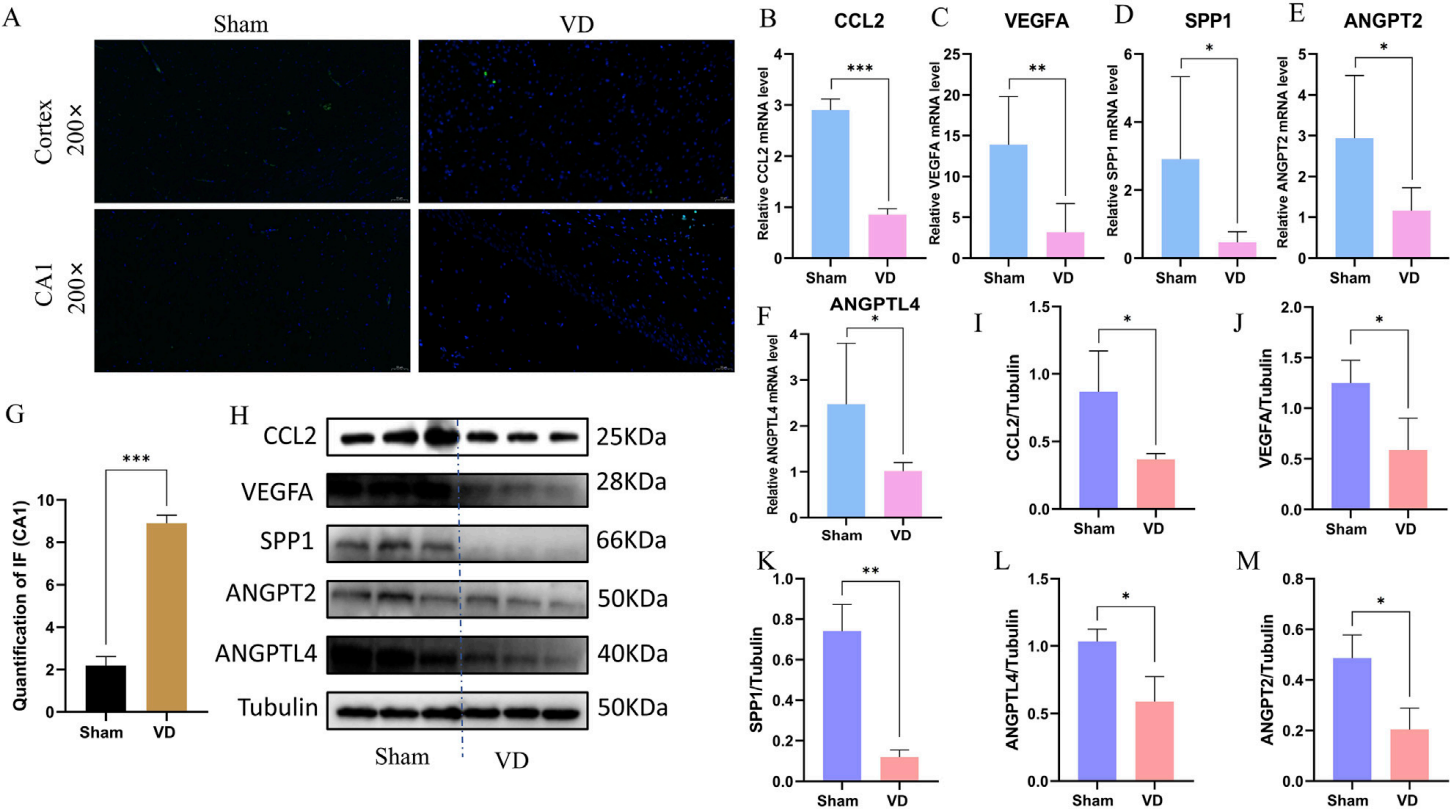
Experimental validation of the key genes *in vivo*. **(A)** Representative images of immunofluorescence. **(B–F)** Expression profile of CCL2, VEGFA, SPP1, ANGPT2, and ANGPTL4 between Sham and VD groups (qPCR; n = 6). *P < 0.05; **P < 0.01; ***P < 0.001. **(G)** Immunofluorescence quantification of CD31. ***P < 0.001. **(H)** WB representative strips of the 5 key genes. **(I–M)** Protein expression level of CCL2, VEGFA, SPP1, ANGPT2, and ANGPTL4 between Sham and VD groups (WB; n = 3). *P < 0.05; **P < 0.01; ***P < 0.001.

To verify whether angiogenesis occurs in the VD model, we performed Immunofluorescence validation on CD31, a classical angiogenesis marker, and found that the expression levels of CD31 in the hippocampus of rats in the VD group were significantly downregulated (Figures 8A,G), indicating the inhibition of angiogenesis phenotype in the VD rats. Quantitative analysis revealed significant downregulation of CCL2, VEGFA, SPP1, ANGPT2, and ANGPTL4 at the transcriptional level in the VD rat model (Figures 8B–F), consistent with our hypothesis. WB analysis further confirmed parallel reductions in protein expression for these five angiogenesis-related factors (Figures 8H–M). These consistent findings at both mRNA and protein levels suggest these molecules may serve as potential diagnostic biomarkers for impaired angiogenesis in VD.

## 4 Discussion

Angiogenesis plays a crucial role in VD pathogenesis and recovery. While pro-angiogenic therapies show promise, specific biomarkers and mechanisms remain unclear. Here, we identified five angiogenesis-related key genes through bioinformatics analysis, revealing their activation of multiple pathways - particularly HIF-1α signaling. SnRNA-seq demonstrated these genes’ predominant expression in microglia, astrocytes, and endothelial cells, with distinct VD-associated expression patterns and cellular distribution imbalances. Functional correlation analyses revealed strong interconnections among these cell populations, suggesting their coordinated involvement in VD progression. GSEA identified 68 relevant pathways, including 8 angiogenesis-linked pathways. These findings were experimentally validated in a rat VD model using BCCAO, confirming the differential expression patterns of identified angiogenic markers. Our results provide new insights into angiogenesis-related molecular mechanisms in VD, offering potential diagnostic markers and therapeutic targets.

CCH is a key pathological driver of VD, primarily through blood-brain barrier (BBB) dysfunction. The BBB, composed of endothelial cells (ECs), pericytes, microglia, and astrocytes, maintains cerebral vascular integrity. In this study, we identified five angiogenesis-related diagnostic genes (*CCL2*, *VEGFA*, *SPP1*, *ANGPT2*, *and ANGPTL4*), predominantly expressed in microglia, astrocytes, and ECs, which critically regulate BBB function. ECs are essential for CNS detoxification, selective protein clearance, and immune cell trafficking. (Sweeney et al., 2018). CCH triggers angiogenic responses, evidenced by elevated angiogenic factors, capillary density, and EC activation in both preclinical and clinical studies (Jing et al., 2015; Shu et al., 2017). Under ischemic conditions, hypoxia serves as the most significant stimulus for angiogenesis (Ek and Englund, 2019). Notably, EC transplantation enhances angiogenesis in ischemic brain tissue (Peng et al., 2018), while endothelial progenitor cell (EPC)-derived microvesicles stimulate neovascularization via mRNA transfer (Deregibus et al., 2007). Our findings reveal that angiogenesis markers are differentially expressed not only in ECs but also in microglia and astrocytes, suggesting coordinated regulation of angiogenesis by these cell types. Correlation analysis demonstrated strong functional interactions among ECs, microglia, and astrocytes, highlighting their collective role in BBB maintenance and angiogenesis modulation. These insights propose novel mechanisms for angiogenesis regulation in VD pathogenesis.

Microglia, the CNS-resident immune cells, critically regulate angiogenesis in both physiological and pathological contexts (Hu et al., 2024). Under normal conditions, they localize near blood vessels and support vascular development. In ischemic stroke, microglia polarize into distinct phenotypes: pro-inflammatory M1 (secreting TNF-α, IL-1β, IL-6) and pro-angiogenic M2 (releasing IL-10, TGF-β, VEGF) (Yenari et al., 2010; Ponomarev et al., 2013). Shifting microglial polarization from M1 to M2 enhances angiogenesis in cerebral ischemia models (Shen et al., 2022; Li et al., 2021). In VD, microglia have become widely activated, displaying a distinct polarization phenotype that leads to their accumulation around neovascular tufts (Yang et al., 2022). This activated microglia induce pathogenic angiogenesis through the secretion of various angiogenic factors and by regulating the status of endothelial cells (Yao et al., 2022). Interestingly, some subtypes of microglia simultaneously promote the regression of neovascularization tufts and normal angiogenesis (Hu et al., 2024). Therefore, modulating the state of microglial activation to improve angiogenesis appears to be a promising therapeutic approach for managing VD. Zhang reported that extracellular vesicles derived from hypoxia-preconditioned microglia facilitate angiogenesis and inhibit apoptosis in stroke models (Zhang et al., 2021). Growing evidence demonstrates that microglial activation critically regulates neurogenesis, angiogenesis, and synaptic plasticity, promoting functional recovery post-cerebral ischemia (Qin et al., 2019; Song et al., 2022). The angiogenic biomarkers identified in this study are all differentially expressed in microglia, indicating their essential roles in vascular remodeling after VD. Targeted interventions aimed at these markers in microglia may offer significant support in the treatment and prevention of VD.

Astrocytes critically regulate angiogenesis and neural tissue reorganization post-brain injury. Following a stroke, they undergo significant morphological and functional alterations, particularly in peri-infarct regions, facilitating neurovascular repair (Allegra Mascaro et al., 2019; Kim et al., 2018). Astrocytes play pivotal roles in vascular repair and neurovascular unit restoration (Freitas-Andrade et al., 2020). Their endfeet extensively envelop cerebral microvessels (Mathiisen et al., 2010), providing structural support and secreting angiogenic factors during vascular development (Reemst et al., 2016). Astrocytes regulate neurovascular coupling by dynamically adjusting blood flow to neuronal activity (Mishra et al., 2016). Hypoxia induces astrocytic VEGF secretion to promote endothelial migration (Rattner et al., 2019), while post-stroke astrocytes release endostatin to modulate angiogenesis (Malik et al., 2020). Reactive astrocytes are key mediators of vascular remodeling after stroke (Williamson et al., 2021), potentially through mitochondrial regulation (Gӧbel et al., 2020). Our study identified astrocyte-specific differential expression of *SPP1* and *ANGPTL4*, highlighting their crucial role in post-stroke angiogenesis. These biomarkers likely mediate vascular remodeling through glial mechanisms, positioning astrocytes as therapeutic targets for vascular recovery. Notably, all five angiogenic markers exhibited strong immune cell interactions, underscoring the coordinated role of microglia and astrocytes (as CNS-resident immune cells) in ischemic stroke and VD pathogenesis.

The five diagnostic markers of angiogenesis identified in this study were all found to be downregulated in the VD model; however, their intrinsic mechanisms of action in VD angiogenesis remain unclear. *CCL2* is a significant chemokine known for its chemotactic activity on monocytes and basophils. Evidence indicates that *CCL2* mediates monocyte recruitment for vascular repair (Low-Marchelli et al., 2013; Zeng et al., 2022; Pan et al., 2020; Lou et al., 2020). *VEGFA* is a growth factor that plays a critical role in angiogenesis and the growth of endothelial cells. It is capable of inducing endothelial cell proliferation, promoting cell migration, inhibiting cell apoptosis, and increasing vascular permeability (Glorioso et al., 2007). Additionally, it participates in the induction of key hypoxia response genes and angiogenesis, such as *HIF1A* (Katsman et al., 2022; Zhang et al., 2014; Brück, 2022). *SPP1* is a secreted phosphoprotein that also functions as a cytokine. In tumors, a substantial number of infiltrating macrophages secrete *SPP1* to stimulate angiogenesis (Chen et al., 2019; Kazakova et al., 2023). Single-cell RNA sequencing has revealed that *SPP1* expression is significantly elevated in microglia and macrophages, with the activation of *SPP1* further enhancing angiogenesis (Wang et al., 2024). *ANGPT2* is a member of the angiopoietin family of growth factors. In the absence of angiogenic inducers, such as *VEGF*, *ANGPT2*-mediated loosening of cell-matrix contacts may lead to apoptosis of endothelial cells, resulting in vascular regression. When acting in conjunction with *VEGF*, *ANGPT2* may facilitate endothelial cell migration and proliferation, thereby serving as a permissive angiogenic signal (Yacyshyn et al., 2009; Yuan et al., 2009). Numerous studies have demonstrated that *ANGPT2* is implicated in BBB leakage and neuronal damage in conditions such as AD and stroke (Van Hulle et al., 2024; Gurnik et al., 2016). ANGPTL4 encodes a glycosylated, secreted protein that can inhibit the proliferation, migration, and tubule formation of endothelial cells, as well as reduce vascular leakage (Beck et al., 2000; Ito et al., 2003; Cazes et al., 2006; Chaube et al., 2023). Experimental evidence indicates that in mouse models of acute ischemic stroke, ANGPTL4 promotes angiogenesis and neurogenesis (Qiu et al., 2021; Spescha et al., 2013; Bouleti et al., 2013), thereby facilitating the recovery of neurological function and cognitive impairment at the earliest possible stage. While *CCL2*, *VEGFA*, *SPP1*, *ANGPT2*, and *ANGPTL4* have established roles in angiogenesis, our study reveals their coordinated dysregulation within neurovascular unit cells in VD. Specifically, we demonstrate that their diagnostic utility as a multi-gene signature, *ANGPT2/ANGPTL4* functional duality under chronic hypoperfusion, and microglial-astrocyte mediation of their effects, offering new cell-specific therapeutic targets.

Although these five angiogenic diagnostic markers are associated with the occurrence of angiogenesis in various diseases, their specific roles and mechanisms in VD remain to be further investigated. Nevertheless, their potential as diagnostic markers for angiogenesis and as targets for treatment and prevention is undeniable. This study acknowledges several limitations. Firstly, the data utilized in this research were sourced from the GEO database, which lacked specific clinical information, thereby constraining the scope of our analysis. Secondly, the sample size obtained from the database was limited, highlighting the necessity for future studies with larger sample sizes to comprehensively elucidate the distinct roles of angiogenesis in VD. Lastly, due to challenges in obtaining VD samples, clinical experiments were not conducted.

## 5 Conclusion

Taken together, transcriptomic bioinformatics analysis identified five diagnostic markers for angiogenesis of VD, and snRNA-seq analysis demonstrated the macro map and brain cell localization of these five molecular markers, which were verified *in vivo*. These five key genes might be used as angiogenesis diagnostic genes for VD and be a novel potential target for diagnosis, treatment, and prevention.

## Data Availability

The original data used in this study are all publicly available. This data can be found here: https://www.ncbi.nlm.nih.gov/geo/.
