# Supplementary material for: Comprehensive analysis and validation of angiogenesis in vascular dementia from the perspective of diagnosis, prevention, and treatment
Source: Front Genet. 2025 Sep 8;16:1646991. doi: 10.3389/fgene.2025.1646991 (PMC12450660; doi:10.3389/fgene.2025.1646991)
Supplement: Supplementary file 2 [file DataSheet1.docx]

**Supplementary material**

**Table S1** Information about the reagents

| Reagent | Manufacturer | Catalog No. | Lot/Batch No. |
| --- | --- | --- | --- |
| SPARKeasy RNA Extraction kit (100T) | Sparkjade | AC0202-B | WPHBS |
| 2×SYBR Green qPCR Mix（With 100×ROX） | Sparkjade | AH0104-B | HUKNU |
| SPARKscript Ⅱ RT Plus Kit (With gDNA Eraser） | Sparkjade | AG0304-B | DTNWH |
| RNase-free and DNase-free | Sparkjade | / | AC1709 |
| 1×PBS | biosharp | BL302A | 24116646 |
| TTC (2%) | Servicebio | G1017 | CR2411017 |

**Figure S2**

Fig. S2: Age characteristics of VD and HC groups (mean ± SD).

**Table S3**

**A** Structured information for public datasets

| **Dataset** | **Search Keywords** | **Organism** | **Initial Samples** | **Excluded (Reason)** | **Final N** |
| --- | --- | --- | --- | --- | --- |
| GSE122063 | "vascular dementia" + "RNA-seq" | Homo sapiens | 80 | 0 | 80 |
| GSE213897 | "single nucleus" + "VD" | Homo sapiens | 15 | 2 (metadata incomplete) | 13 |

**B** Gender information in GSE122063

| group | male | female | total |
| --- | --- | --- | --- |
| VD | 20（55.56%） | 16（44.44%） | 36 |
| HC | 24（54.55%） | 20（45.45%） | 44 |

**C** Gender information in GSE213897

| group | male | female | total |
| --- | --- | --- | --- |
| VD | 3（37.5%） | 5（62.5%） | 8 |
| HC | 2（40%） | 3（60%） | 5 |

**Table S4**  Primer sequence

| **Number** | **Primer** | **Sequence(5'to3')** |
| --- | --- | --- |
| 1 | Rat-VEGF F | CATGGCAGAAGGAGACCAGA |
| 2 | Rat-VEGF R | CACCAGGGTCTCGATTGGA |
| 3 | Rat-CD31 F | GGAGGTATCGAATGGGCAGA |
| 4 | Rat-CD31 R | CCGAGACTGAGGAATGACGA |
| 5 | Rat-ANGPT2 F | GTAGCCCCTTCCCACATCAG |
| 6 | Rat-ANGPT2 R | CTTTGGTTGGTTAGCGGTGC |
| 7 | Rat-HIF-1α F | CGCAGTGTGGCTACAAGAAA |
| 8 | Rat-HIF-1α R | AGGCTGTGTCGACTGAGAAA |
| 9 | Rat-GAPDH F | CAAGGCTGAGAATGGGAAGC |
| 10 | Rat-GAPDH R | GAAGACGCCAGTAGACTCCA |
| 11 | Rat-SPP1 F | CCACAGTCGATGTCCCTGAC |
| 12 | Rat-SPP1R | GGCAACTGGGATGACCTTGA |
| 13 | Rat-CCL2F | TGATCCCAATGAGTCGGCTG |
| 14 | Rat-CCL2R | TGGACCCATTCCTTATTGGGG |
| 15 | Rat-ANGPTL4F | CAGCTGCGATCTACAGGCTT |
| 16 | Rat-ANGPTL4R | CAACGCTAGGACTTCGGGAC |

**Figure S5**


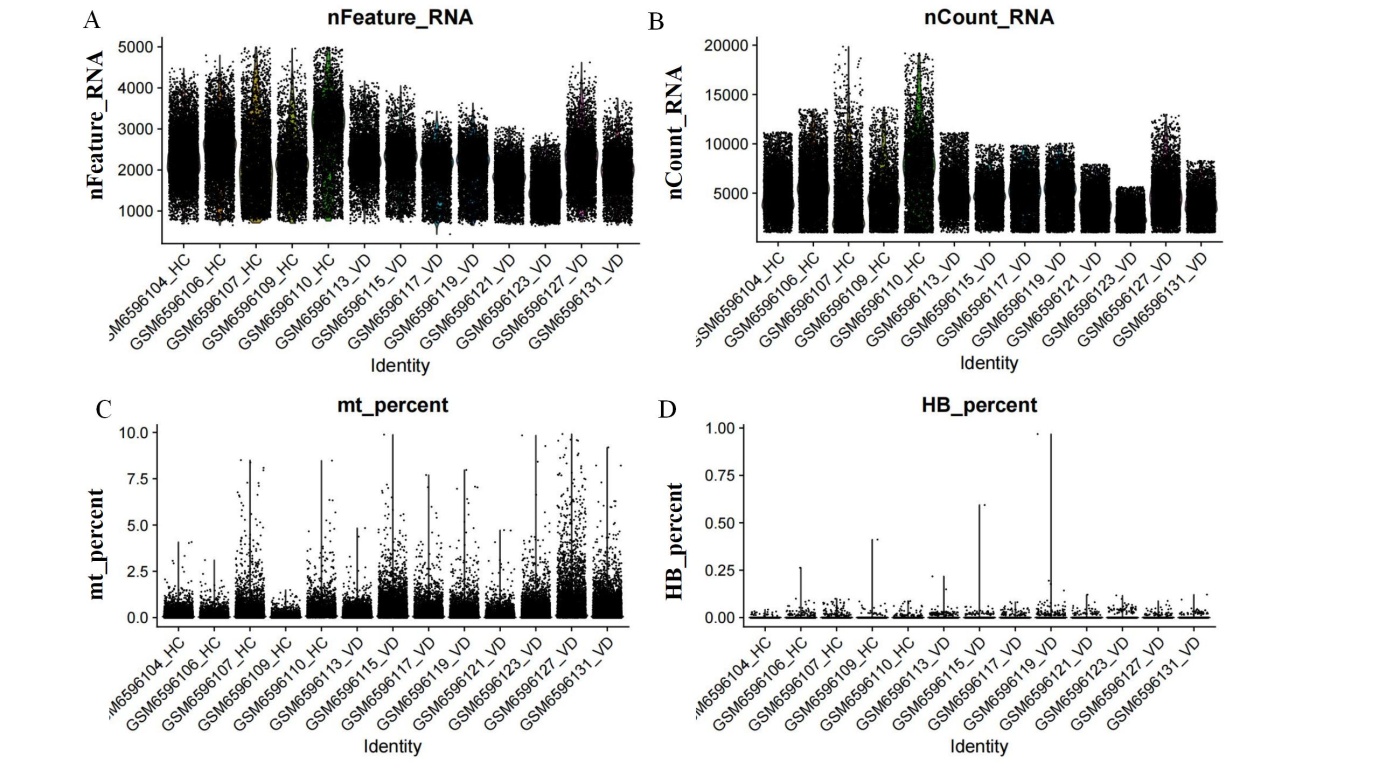


Fig. S5: SnRNA-seq data after quality control. (A) nFeature_RNA. (B) nCount_RNA. (C)mt_percent. (D)HB_percent.

**Figure S6**

**
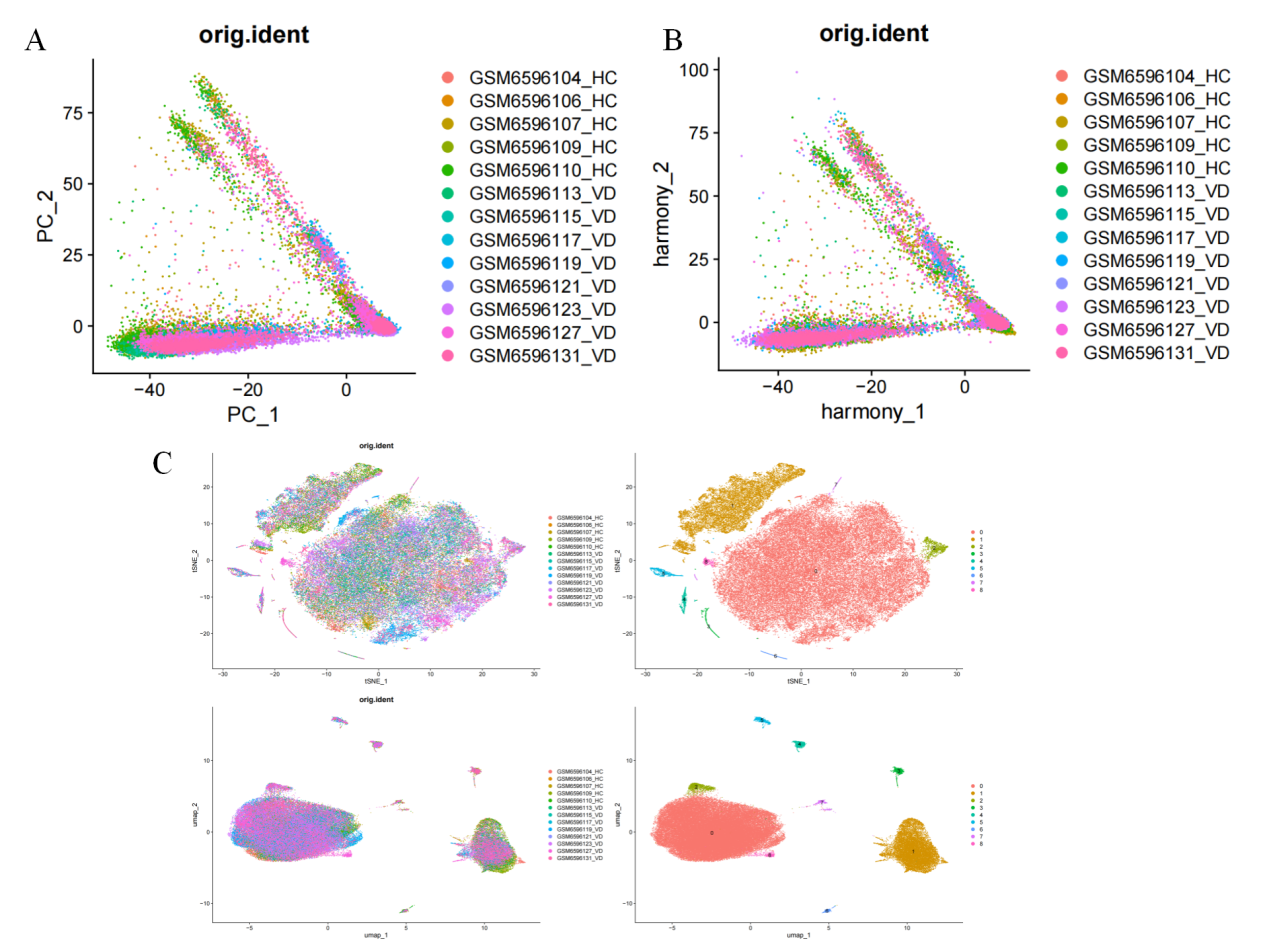
**

Fig. S6: (A) PCA plot before harmony. (B) PCA plot after harmony. (C) UMAP/t-SNE plot after harmony.

**Figure S7**


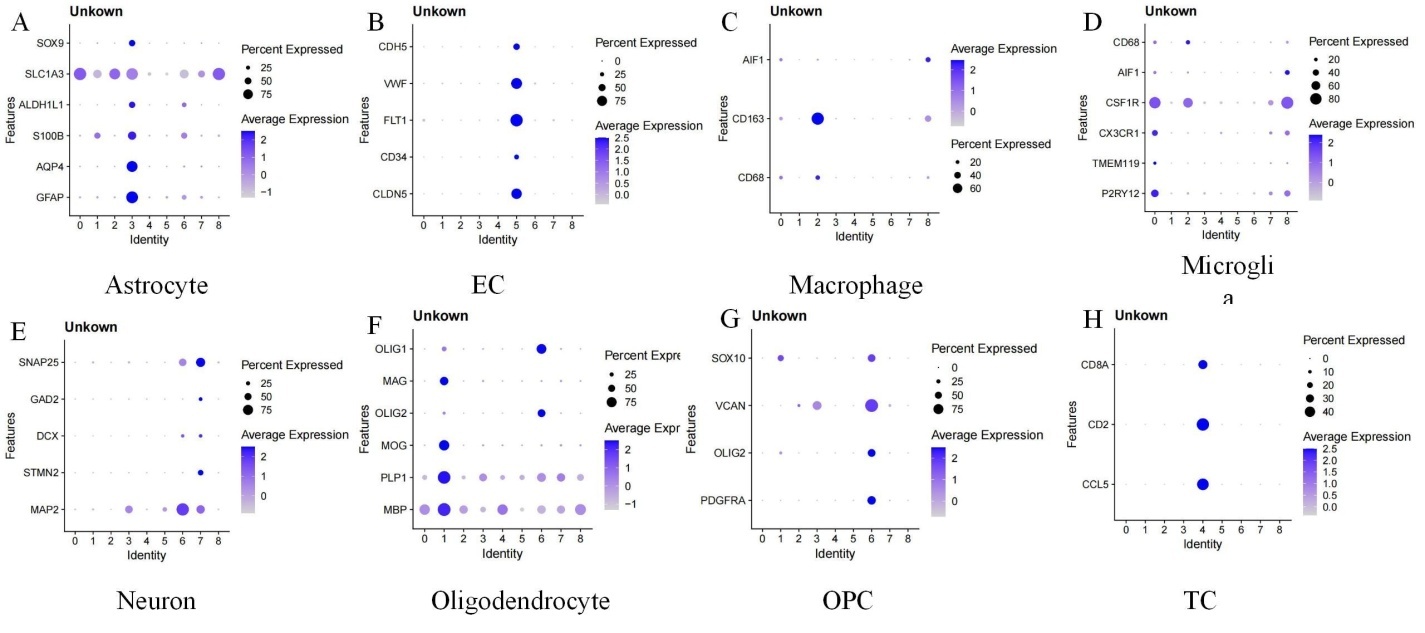


Fig. 7: The mark gene validation plots for each cell cluster.

**Figure S8**

B


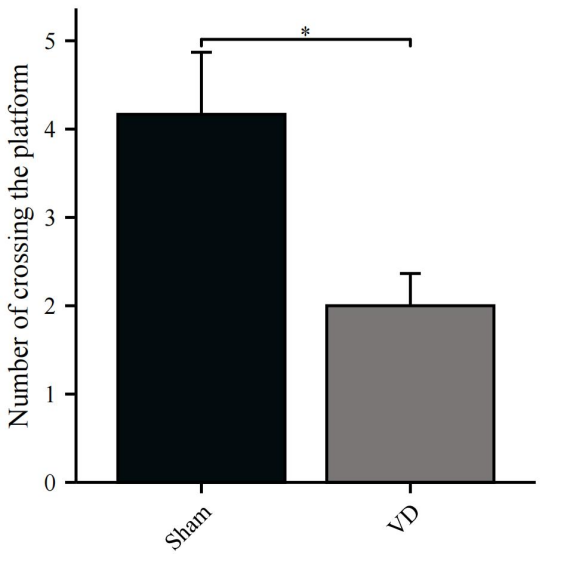

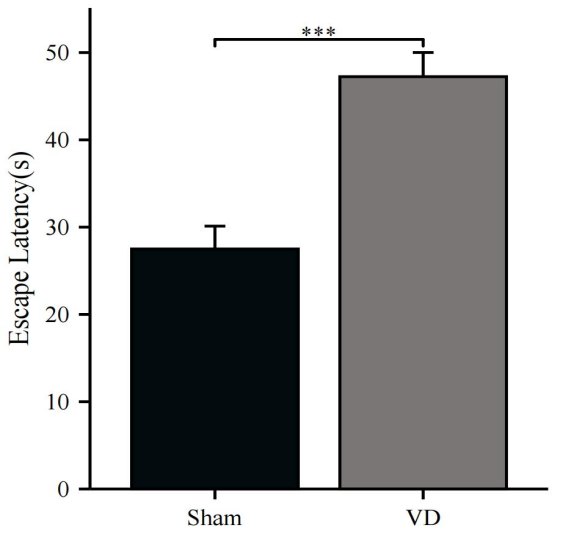


A

Fig. S8: (A) Comparison of escape latency between the Sham and VD groups (n = 6). (B) Comparison of the numbers of crossing platforms between the Sham and VD groups (n = 6). *P < 0.05; **P < 0.01; ***P < 0.001.
